# Frequency of five disease-causing genetic mutations in a large mixed-breed dog population (2011–2012)

**DOI:** 10.1371/journal.pone.0188543

**Published:** 2017-11-22

**Authors:** Sharon Zierath, Angela M. Hughes, Neale Fretwell, Mark Dibley, Kari J. Ekenstedt

**Affiliations:** 1 Veterinary and Biomedical Sciences Department, College of Veterinary Medicine, University of Minnesota, Saint Paul, Minnesota, United States of America; 2 Mars Veterinary, Vancouver, WA, United States of America; 3 Department of Basic Medical Sciences, College of Veterinary Medicine, Purdue University, West Lafayette, Indiana, United States of America; University of Sydney Faculty of Veterinary Science, AUSTRALIA

## Abstract

**Background:**

A large and growing number of inherited genetic disease mutations are now known in the dog. Frequencies of these mutations are typically examined within the breed of discovery, possibly in related breeds, but nearly always in purebred dogs. No report to date has examined the frequencies of specific genetic disease mutations in a large population of mixed-breed dogs. Further, veterinarians and dog owners typically dismiss inherited/genetic diseases as possibilities for health problems in mixed-breed dogs, assuming hybrid vigor will guarantee that single-gene disease mutations are not a cause for concern. Therefore, the objective of this study was to screen a large mixed-breed canine population for the presence of mutant alleles associated with five autosomal recessive disorders: hyperuricosuria and hyperuricemia (HUU), cystinuria (CYST), factor VII deficiency (FVIID), myotonia congenita (MYC) and phosphofructokinase deficiency (PKFD). Genetic testing was performed in conjunction with breed determination via the commercially-available Wisdom Panel^TM^ test.

**Results:**

From a population of nearly 35,000 dogs, homozygous mutant dogs were identified for HUU (n = 57) and FVIID (n = 65). Homozygotes for HUU and FVIID were identified even among dogs with highly mixed breed ancestry. Carriers were identified for all disorders except MYC. HUU and FVIID were of high enough frequency to merit consideration in any mixed-breed dog, while CYST, MYC, and PKFD are vanishingly rare.

**Conclusions:**

The assumption that mixed-breed dogs do not suffer from single-gene genetic disorders is shown here to be false. Within the diseases examined, HUU and FVIID should remain on any practitioner’s rule-out list, when clinically appropriate, for all mixed-breed dogs, and judicious genetic testing should be performed for diagnosis or screening. Future testing of large mixed-breed dog populations that include additional known canine genetic mutations will refine our knowledge of which genetic diseases can strike mixed-breed dogs.

## Introduction

The modern domesticated dog is a highly variable species, with purebred individuals representing nearly 500 different breeds globally [1]. In the last 100–300 years of modern breed formation [2], the development of strict breed standards led to aggressive inbreeding tactics, and this amplified the prevalence of autosomal recessive genetic disorders. Once breeds were established and breed stud books closed, it became standard among institutions such as the American Kennel Club that both the dam and sire had to be registered purebreds in order to register their offspring as purebred [3]. These practices led to closed genetic pools that are now relatively small compared to the species as a whole.

Many monogenic disorders have been identified in specific purebred canine breeds. These are often selected for inadvertently because of linkage disequilibrium; when breeders select for specific traits, occasionally “hitchhiker” genes, which are closely physically linked to the selected trait on the chromosome, will be inherited together with the intended trait. For example, selective pressure on coat pattern in Dalmatians for larger, more pronounced spots has led to a relatively fixed phenotype within the breed. Based on segregation analysis, it appears that a modifier of spot size is closely linked to the locus for hyperuricosuria and hyperuricemia (HUU) [4], a syndrome resulting in elevated levels of uric acid in the blood and urine, which is fixed in the Dalmatian breed. Indeed, all purebred, non-backcrossed, Dalmatian dogs are homozygous for two copies of the *SLC2A9* gene mutation associated with HUU [5].

There are several hundred naturally-occurring inherited canine diseases, a substantial portion of which show simple Mendelian inheritance; molecular genetic tests are available for well over 100 of these diseases [6–8]. Such disorders are obviously of significant concern when selecting a breeder or a breed line for a purebred puppy. Conversely, this level of concern is typically not present when selecting a mixed-breed puppy or dog, because the pet-owning public as well as veterinarians often assume such dogs will exhibit hybrid vigor and not be affected by inherited monogenic (Mendelian) disorders. This assumption could potentially lead to a missed diagnosis, particularly for those single-gene disorders of higher frequency and for those that are observed in more than one breed.

Monogenic disorders are expected to occur less frequently within a wider gene pool (e.g., mixed-breed dogs) than within narrow purebred populations. It is well known that specific genetic disorders can occur with a greater frequency among some pure breeds compared to others, and that a single breed can manifest certain genetic disorders at a very high prevalence. A genetic disorder that is carefully considered in treating a purebred dog might not be expected in a mixed-breed dog of unknown origins. McGreevy and Nicholas [9] pointed out that an F1 dog (first generation offspring from two different purebred parents) has a far lower chance of exhibiting the genetic disorders common to the parental breeds, and this can be extrapolated to dogs that are even more “mixed” than 50/50. However, a genetic disease could still have been introduced in early generations of a mixed-breed dog and should not automatically be dismissed from a diagnostic rule-out list. The practitioner is therefore left unsure when to consider genetic disease as a diagnosis in a mixed-breed dog; certainly some genetic diseases must occur with such a low frequency across all canids that specific concern in mixed-breed dogs is negated; however, others may have a high enough frequency that owners and attending veterinarians should pursue genetic testing. Therefore, the present study aims to examine, for the first time, a small number of genetic disease mutations in a large number of mixed-breed dogs in order to determine which diseases, if any, merit consideration, even in mixed-breed dogs.

Mars Veterinary, a division of Mars, Inc., produces a line of commercially available Wisdom Panel^TM^ genetic tests to identify the approximate ancestral breeds of individual mixed-breed dogs, based on the work of Parker *et al*. [10]. Many tens of thousands of dogs have been sampled in a primarily North American pool, representing an excellent cohort of mixed-breed dogs. In addition to testing breed makeup, certain Wisdom Panel^TM^ tests also screen for five previously identified and published Mendelian disorder mutations: 1) hyperuricosuria, 2) cystinuria, 3) factor VII deficiency, 4) myotonia congenita, and 5) phosphofructokinase deficiency.

Hyperuricosuria and hyperuricemia (HUU) is a recessive Mendelian disorder attributed to a *SLC2A9* gene missense mutation (a single base change, c.616G>T; cysteine changed to phenylalanine) that causes a build-up of uric acid in the urine, and predisposes the affected individual to the formation of bladder and kidney stones [5]. This mutation was originally reported in three unrelated breeds: the Dalmatian, English Bulldog, and Black Russian Terrier [5]. Subsequently, the same mutation was identified in additional breeds with varying frequency: American Staffordshire Terrier, Australian Shepherd, Giant Schnauzer, German Shepherd Dog, Labrador Retriever, Large Munsterlander, Parson (Jack) Russell Terrier, Pomeranian, South African Boerboel, Weimaraner [11], and Spanish Water Dog [12]. It was also very recently found in six additional European breeds: Danish-Swedish Farmdog, German Hunting Terrier, Kromfohrländer, Lagotto Romagnolo, Spaniel de Pont-Audemer, and Swedish Vallhund [13]. Not all of these breeds were included/tested for by the Wisdom Panel^TM^ test at the time of this study (specifically the Boerboel, Danish-Swedish Farmdog, German Hunting Terrier, Kromfohrländer, Lagotto Romagnolo, and Spaniel de Pont-Audemer) due to their rarity in North America, and therefore would not have been identified in any dog submitted for testing. The lack of a close relationship and the diverse backgrounds of these twenty breeds suggest that the *SLC2A9* mutation originated in an ancestor pre-dating the development and/or closure of the studbook for these breeds. In fact, a terrier-type dog skeleton recovered from the *Mary Rose*, a Tudor warship which sank in 1545, was shown to be heterozygous for this mutation [14], showing that it clearly predates the development of most modern dog breeds.

Cystinuria (CYST) is another inherited renal disease, characterized by defective amino acid reabsorption and cysteine urolithasis [15]. The first identified canine mutation associated with CYST was an autosomal recessive Mendelian nonsense mutation in exon 2 of *SLC3A1* in Newfoundlands and Landseers (which are very closely related); a single base C-to-T change (663C>T) was responsible for the premature stop codon [15]. Subsequently, additional different CYST-associated mutations have been identified in *SLC3A1* in other breeds of dog (Labrador retriever and Australian Cattle Dogs, with autosomal-recessive and autosomal-dominant modes of inheritance, respectively) [16]. To date, no other breed has been shown to have the Newfoundland/Landseer C633T mutation, which is the one tested for in the present study. This mutation would therefore, in theory, be of less concern in a wider population of complex mixed-breed dogs, due to the very narrow profile of affected breeds.

Factor VII deficiency (FVIID) is a clotting disorder resulting from a lack of proconvertin (clotting factor VII) in the blood, which typically results in mild bleeding episodes and bruising; the coagulopathy may go entirely unnoticed until detected via coagulation screening tests or excessive bleeding episodes brought on by surgery or trauma [17,18]. FVIID is inherited in an autosomally recessive manner; a missense mutation in the *F7* gene (c.407G>A), which changes a glycine to glutamic acid, was first reported in research colony Beagles, then in pet Beagles [17]. The same mutation was then identified in Alaskan Klee Kai dogs, which had severe bleeding abnormalities [18], and in the Scottish Deerhound [13]. A recent study has now additionally documented this same mutation in the American Foxhound, Finnish Hound, German Wirehaired Pointer, Irish Water Spaniel, Japanese Spitz, Miniature Schnauzer, Papillon/Phalene, Sealyham Terrier, and Welsh Springer Spaniel [13]. Finally, there are unsubstantiated reports that this mutation is also found in Airedale Terriers and Giant Schnauzers. Just as with the *SLC2A9* mutation in HUU, the diverse breeds shown to carry the identical *F7* mutation indicate not only that this mutation predates many/most dog breed development, but also that it should be considered as a rule-out even in mixed-breed dogs, particularly since expression of the FVIID gene mutation is not always consistent from one breed to another, i.e., this mutation has been associated with minor bleeding complications in Beagles but severe hemorrhagic episodes in the Alaskan Klee Kai [17,18].

Myotonia congenita (MYC) is a relatively rare disorder perhaps made most famous by “fainting goats”; it is characterized by delayed relaxation of muscle after sudden, forceful contractions, which create animals that are stiff-legged, or “fainting” due to the acute severe muscle stiffness and continuous contraction of voluntary muscles that can cause them to fall [19]. In Miniature Schnauzers, an autosomal recessive form of MYC was found to be associated with a missense mutation in the *CLCN1* (formerly the *ClC-1*) gene, the skeletal muscle voltage-gated chloride channel. The C to T transition replaces a threonine residue with a methionine (C>T; T268M) [20]. One European-based study examined this mutation in 70 different additional breeds and failed to identify it in any of them; it should be noted, though, that most breeds were represented by only one or a very few individuals [21]. However, one case report of a single Jack Russell Terrier with MYC determined that this dog had a spontaneous mutation (its parents were clear of the mutation) identical to that seen in the Miniature Schnauzer [22], suggesting that this particular mutation may be seen in other breeds through random chance (rather than identity by descent as an ancient mutation). It is still entirely unclear how prevalent this mutation could be among all canine populations. It should be noted that an additional MYC-associated mutation has been identified in the *CLCN1* gene in Australian Cattle Dogs [23] and Border Collies [13]; however, this is an insertion mutation at a different location in the gene from the Miniature Schnauzer. The present study tested only for the Miniature Schnauzer mutation.

Finally, phosphofructokinase deficiency (PFKD) is another autosomal recessive metabolic disorder characterized in dogs primarily by hemolytic crisis [24]. Affected dogs can also have exertional myopathy, observed by their owners as exercise intolerance [25,26]. A G to A transition mutation (c.2228G>A), replacing tryptophan with a stop codon, has been described in the *PFKM* gene in canids; this same nonsense mutation has been identified in American Cocker Spaniels [27], English Springer Spaniels [25,28], and Whippets [26]. While Cocker Spaniels and Springer Spaniels are relatively similar breeds, Whippets represent a different genetic background; thus one might expect to see this mutation dispersed more widely amongst mixed-breed dogs. Recently, an additional mutation in the same *PFKM* gene was identified in Wachtelhund dogs with PFKD. This mutation, however, is a missense mutation at a different genetic location [24], indicating that it arose separately from the one described in American Cocker Spaniels, English Springer Spaniels, and Whippets. The present study tested only for the Spaniel/Whippet mutation.

The aim of the present study was to determine the frequencies of these five specific Mendelian disorder alleles and affected genotypes in a large sample population of mixed-breed dogs. We hypothesized that: 1) the mutations associated with HUU, FVIID, and PFKD would be observed more commonly in mixed-breed dogs, based on their being more ancient mutations detectable in multiple, unrelated breeds; and 2) the mutations associated with CYST and MYC would be observed more rarely in a mixed-breed dog population and might only be observed when a dog has specific breeds in its background known to have these mutations. Establishing the frequencies of these mutations in mixed-breed dogs will allow increased understanding of the disorders that occur in mixed-breed dogs and will provide diagnostic guidance to both owners and veterinarians.

## Materials and methods

A data set of 34,324 individual dog samples was collected and analyzed by Mars Veterinary under the commercially available Wisdom Panel^TM^ brand tests. Samples were collected over a six month period between December 2011 and May 2012. The cohort consisted primarily of North American dogs, which were mainly United States-based, although some were also submitted from Canada. A small percentage, 2.66% (n = 912) and 1.13% (n = 389), of this population are from the United Kingdom and Australia, respectively. Purebred dogs are occasionally submitted for Wisdom Panel^TM^ testing, whether their purebred status is known, suspected, or unknown when submitted. All purebred dogs were removed from the dataset, except for a very small number, which are technically purebred; these dogs are retained because their genetic signatures suggested disparate continental origins (for example: UKBeagle vs. USBeagle) or breed signatures that suggest non-AKC breeds that overlap significantly, but not entirely, with AKC breeds (for example: German Shepherd and White Swiss Shepherd).

Samples submitted for Wisdom Panel^TM^ testing are either whole blood or cheek swabs. IACUC approval was not required for this work, as it consisted only of mining data that was acquired via a voluntarily-utilized commercially available test. Cheek swab samples were performed by the owners at home, and blood samples were obtained by the dogs’ attending veterinarians.

DNA was extracted from blood or cheek swab samples using standard protocols. Breed status of each dog’s sample was evaluated after genotyping the Wisdom Panel^TM^ test’s 321 single nucleotide polymorphisms (SNPs) on the Sequenom MassARRAY platform. SNP data was analyzed by a proprietary computer algorithm that assigns the best match to the familial breed makeup of each dog from a purebred database within a certain confidence interval. In more detail, the breed makeup of each dog is determined by a complex, proprietary series of algorithms that compare the DNA of a sample dog with over seven million possible breed combinations, sorted by a pedigree tree going back three generations (parent, grandparent, and great-grandparent). The patented comparison method genotypes the 321 SNPs across 25 chromosomes for each submitted dog, and then compares those SNP genotypes to a reference pool of purebred dogs consisting of more than 10,000 dogs representing just over 190 breeds (the exact number varied from 191 to 202 depending on which Wisdom Panel^TM^ test was performed). A pedigree tree of eight great-grandparent dogs was assigned for each dog. The resulting pedigree tree for a sampled dog can range anywhere from a purebred result (all great-grandparent dogs representing the same breed) to a very complex result (all eight great-grandparent dogs representing different breeds).

Dogs were additionally genotyped for the five disease-associated SNPs (HUU, CYST, FVIID, MYC, and PFKD) on the Sequenom platform. Data retrieved for the present study for each sample included: a unique identification number, all eight great-grandparent breeds, and genotype status for each of five Mendelian disorders: HUU, CYST, FVIID, MYC, and PFKD. Each of the five disease genotypes—the actual published SNP mutations—was determined using a TaqMan-based RT-PCR assay protocol. All sampled dogs were assigned a genotype for each of the five disorders: two copies (homozygous) for the alternate/mutated allele; one copy (heterozygous) for the alternate/mutated allele; or no copies of the alternate/mutated allele (homozygous for the wild type allele). Some dogs were not successfully tested for all five disorders, therefore the total number of dogs tested for each disorder varied slightly. The frequency of the alternate/mutated allele, the frequency of the heterozygote (carrier) genotype, and the frequency of the genetically-susceptible (homozygous, presumably affected) phenotype were all calculated separately for each disorder within this large, mixed-breed population via allele counting, and were reported as percentages with a binomial 95% confidence interval generated using the Clopper/Pearson method [29]. Phenotype frequencies assume perfect genotype-phenotype correlation; follow-up with the small number of genetically-susceptible dogs was not undertaken to verify affected disease status due to the retrospective nature of this study. Pairwise Fisher tests were conducted, with Bonferroni-Holm correction for multiple comparisons, separately for each of the following, comparing each disorder’s frequency to all other frequencies within the same category: 1) frequency of the alternate/mutated allele, 2) frequency of the carrier genotype, and 3) frequency of the genetically-susceptible (homozygous) genotype. R (version 3.2.3) [30] was used to perform all statistical tests, and a p-value less than or equal to 0.05 was considered significant.

Finally, certain breed trends were expected among the various disorders; for example, dogs with Dalmatian and/or English Bulldog ancestry in their background were expected to be over-represented in the HUU-affected group. Therefore, genetically-susceptible individuals for each disorder were evaluated according to breed makeup and any breed trends were noted.

## Results

A total of 34,324 mixed-breed dogs were analyzed for breed makeup (S1 Table) and presence of five Mendelian disorders. Not all dogs were successfully screened for each of the five disorders, therefore, the cohort total for each diseases is slightly different.

The disease-associated mutations with the highest frequency among this large cohort were those for HUU and FVIID (Table 1). Alternate/mutated alleles for PFKD were observed only in the heterozygous state, and the other two were virtually (CYST) or entirely (MYC) absent.

**Table 1:** Observed frequencies of alternate/mutated alleles by disorder in a large, mixed-breed canine population.

Disorder	Gene	Mutation	Number Tested	Number of Heterozygotes	Percentage of Heterozygotes	95% CI	Number of Homozygotes	Percentage of Homozygotes	95% CI	Percentage of Alternate/Mutated Alleles	95% CI
Hyperuricosuria and hyperuricemia (HUU)	*SLC2A9*	c.616G>T	34,118	1,517	4.45% (a)	4.23% - 4.67%	57	0.167% (a)	0.12% - 0.22%	2.39% (a)	2.28% - 2.51%
Cystinuria (CYST)	*SLC3A1*	c.633C>T	34,104	1	0.00293% (d)	0.00% - 0.016%	0	0% (b)	0.00% - 0.011%	0.00146% (d)	0.00% - 0.008%
Factor VII Deficiency (FVIID)	*F7*	c.407G>A	34,031	288	0.846% (b)	0.75% - 0.95%	65	0.191% (a)	0.15% - 0.24%	0.614% (b)	0.56% - 0.68%
Myotonia Congenita (MYC)	*CLCN1*	C>T; T268M	33,761	0	0% (d)	0.00% - 0.011%	0	0% (b)	0.00% - 0.011%	0% (d)	0.00% - 0.005%
Phosphofructokinase Deficiency (PFKD)	*PFKM*	c.2228G>A	34,112	19	0.0557% (c)	0.034% - 0.089%	0	0% (b)	0.00% - 0.011%	0.0278% (c)	0.02% - 0.04%

Frequencies are reported as percentages. Pairwise Fisher tests (p-values provided in S2 Table) were conducted between the five disorders for each type of frequency: percentage of heterozygous dogs, percentage of homozygous dogs, and percentage of alternate/mutated alleles. Within each column, the letter indicates this value is statistically significantly different from all other different letters in that column at p ≤ 0.05. Heterozygotes = carrier dogs; Homozygotes = genetically-susceptible dogs; CI = confidence interval.

The alternate/mutated *SLC2A9* allele associated with HUU was present with an allelic frequency of 0.0239 (2.39%, 95% CI 2.28% - 2.51%), and a homozygous mutated (genetically-susceptible) genotype frequency of 0.00167 (n = 57; 0.167%, 95% CI 0.12% - 0.22%) among the total population tested (n = 34,118). The heterozygous genotype was present with a frequency of 0.0445 (n = 1,517; 4.45%, 95% CI 4.23% - 4.67%). Of the 57 dogs homozygous for the alternate/mutated HUU allele, 38 of them had a breed known to carry the HUU allele (from the 20 previously-reported breeds) present for at least two great-grandparents reported in their ancestry that could have contributed the alternate/mutant allele. Five of the 57 alternate/mutant HUU allele homozygous dogs had a previously-reported HUU-carrying breed identified as a single great-grandparent in their ancestry. Unsurprisingly, the most commonly observed HUU-carrying breeds amongst the 57 homozygous dogs in this mixed-breed population were the Dalmatian (identified at the great-grandparent level or closer in 9 of the dogs), English Bulldog (identified at the great-grandparent level or closer in 7 of the dogs), and the American Staffordshire Terrier (identified at the great-grandparent level or closer in 25 of the dogs). Some dogs had detectable signatures from more than one of these three breeds (n = 3), e.g., American Staffordshire Terrier and Bulldog or American Staffordshire Terrier and Dalmatian. A very small number of HUU alternate/mutant allele-homozygous dogs had others of the previously-reported HUU-carrying breeds detected, including Labrador Retriever, Australian Shepherd, and German Shepherd (each breed observed in three dogs), and Weimaraner, which was observed in one dog. Of these, one dog had both Australian Shepherd and Weimaraner detected in its ancestry, and a second dog had both Australian Shepherd and German Shepherd detected in its ancestry. Fourteen homozygous alternate/mutant allele dogs did not have any known HUU-carrying breeds detected at all in their recent ancestry. Of the 57 homozygous dogs, eight of them did not have any breed detected above the great-grandparent level, indicating these dogs had very complex ancestry.

The sample population for FVIID consisted of 34,031 dogs, and 65 were identified as homozygous for the alternate/mutated allele, yielding a genetically-susceptible frequency of 0.00191 (0.191%; 95% CI 0.15% - 0.24%). The heterozygous carriers numbered 288 dogs, resulting in a carrier rate of 0.00846 (0.846%; 95% CI 0.75% - 0.95%), and an overall alternate/mutated allele frequency of 0.00614 (0.614%; 95% CI 0.56% - 0.68%) in this sample cohort. Of the 65 dogs homozygous for the alternate/mutated FVIID allele, only 12 had a breed known to carry the FVIID allele (from the 14 previously-reported breeds) present in at least two identified great-grandparents. Three of the 65 affected dogs had a previously-reported FVIID-carrying breed reported for only a single great-grandparent. The most commonly observed FVIID-carrying breed amongst the 65 homozygous dogs in this mixed-breed population was the Beagle, which was identified at the great-grandparent level or closer in 13 of the dogs. Other known FVIID-carrying breeds were rarely represented; at the great-grandparent level or closer, they included: Miniature Schnauzer (observed in two dogs), and American Foxhound, Scottish Terrier, and Welsh Springer Spaniel (each observed in one dog). Three dogs had detectable breed signatures from more than one of these breeds, e.g., grandparent Beagle and grandparent American Foxhound, or grandparent Beagle and grandparent Miniature Schnauzer. Fifty dogs did not have any known FVIID-carrying breeds detected in their recent ancestry. Of the 65 homozygous dogs, 11 of them did not have any breed detected above the great-grandparent level, indicating these dogs had very complex ancestry.

Of the dogs tested for CYST (n = 34,104), only one was found to be heterozygous for the alternate/mutated allele, and no homozygous affected dogs were observed. A single great-grandparent Newfoundland was detected in this carrier dog, likely explaining the inheritance of the alternate/mutated allele. The allelic frequency for the alternate/mutated allele in this large, mixed-breed population was 0.0000146 (0.00146%; 95% CI 0.00% - 0.008%) and a carrier rate of 0.0000293 (0.00293%; 95% CI 0.00% - 0.016%).

Within the sample population tested for the PFKD alternate/mutated allele (n = 34,112), 19 dogs were identified as heterozygous for the mutation. No dogs were homozygous for the mutation. The allelic frequency of the alternate/mutated allele attributed to PFKD was 0.000278 (0.0278%; 95% CI 0.02% - 0.04%) in this cohort, and the carrier rate was 0.000557 (0.0557%; 95% CI 0.034% - 0.089%). Among the 19 carrier dogs, only one dog was identified with an English Springer Spaniel grandparent, leaving 18 dogs with no recent ancestor breeds detected for which this specific mutation has previously been reported.

The population tested for the mutated *CLCN1* gene related to MYC (n = 33,761) was entirely homozygous for the wild-type allele; no copies of the alternate/mutated allele were observed in this cohort of mixed-breed dogs, giving an alternate/mutated allele frequency of 0.

Pairwise Fisher tests for the alternate/mutated allele frequency in this population indicate that the alternate/mutated allele of HUU is significantly more prevalent compared to the other four, the alternate/mutated allele of FVIID is significantly more prevalent than the remaining three, and the alternate/mutated allele of PFKD is significantly more prevalent than CYST or MYC. This pattern was identically observed for the carrier/heterozygous genotype frequency. Pairwise Fisher tests for the genetically-susceptible state (presumably affected dogs) indicated that both HUU and FVIID were significantly more frequent than the other three disorders.

Lastly, out of the 34,324 dogs, 5.29% (n = 1815) were heterozygous for at least one of the tested variants, and 10 dogs were heterozygous for two disorders. No dog had alternate/mutated alleles from more than two of the tested disorders. One dog was heterozygous for the HUU mutation and also heterozygous for the PFKD mutation, and nine dogs were heterozygous for the HUU mutation and also heterozygous for the FVIID mutation. Two dogs were homozygous for the FVIID mutation and also heterozygous for the HUU mutation. There were 122 dogs that were homozygous and genetically-susceptible for a disorder (57 for HUU and 65 for FVIID); no dogs were homozygous for more than one disorder.

## Discussion

This study offers a unique dataset in which to examine frequencies of known genetic diseases within a very large, primarily North American population of mixed-breed dogs that spans the entire United States, with small contributions from Canada, and with additional small numbers of dogs from Australia and the UK. This sample population allows the mixed-breed dog owner and the practitioner a realistic, statistical measure of when to consider specific genetic diseases and which genetic diseases are highly unlikely to occur in a mixed-breed dog.

Heterozygosity (“carrier” status) for HUU was observed at a statistically significantly higher frequency than for any of the other four diseases (n = 1,517 out of 34,118 or 4.45% compared to the next highest, 0.85% for FVIID). Twenty breeds have been reported to carry the HUU alternate/mutant allele. In our study, fifty-seven dogs were found to be homozygous for the HUU alternate/mutated allele, and many of them had, as expected, Dalmatian, English Bulldog, and/or American Staffordshire Terrier ancestry. A low number of other breeds previously-reported to carry the HUU allele were also observed in the affected dogs. However, for fourteen homozygous mutant HUU dogs, none of the twenty previously-reported breeds was detected; similarly, in 5 dogs, only one great-grandparent breed was detected from the twenty previously-reported breeds. This could be due to the failure of the algorithm to detect very small contributions of such breeds (e.g., Dalmatian, English Bulldog, etc.) from further back than the great-grandparent level, but it also strongly suggests that additional breeds will eventually be shown to carry the alternate/mutant HUU allele. Breeds that were observed more frequently in these HUU-unexplained mixed-breed dogs included several others from the “guard” type, including the American Bulldog, French Bulldog, Staffordshire Bull Terrier, Bull Terrier, and Miniature Bull Terrier; it may be that eventually these breeds will be confirmed to carry the HUU alternate/mutant allele, although this study is not designed to answer that question. Considering the close relationship of the American Staffordshire Terrier and the English Bulldog breeds to other guard-type breeds, such as those detected, it is reasonable to consider HUU in a clinical setting for any mixed-breed dog with guard-type ancestry. Based on the results of this study, the practitioner may consider evaluating the concentration of uric acid in the urine of any guard-type, mixed-breed dog, similar to the approach taken with Dalmatian dogs. Alternatively, and more proactively, the practitioner may choose to recommend performing the genetic test specific to HUU, available through several testing laboratories and increasingly tested for in panels included with mixed-breed genetic testing, in order to assess the likelihood of the patient developing this problem. Because of the high prevalence of this alternate/mutated allele even among a mixed-breed population, and because of the diverse background of known breeds carrying this alternate/mutant allele, testing for HUU, either clinically or genetically, should not be excluded on the grounds of a dog’s mixed-breed status; conversely, HUU should remain on the practitioner’s rule-out list, when clinically appropriate, for all mixed-breed dogs. This point is shown particularly dramatically in our detection of homozygous HUU-mutant status for eight dogs with highly complex genetic ancestry; i.e., in even the most highly-mixed dogs, homozygous mutants can occur.

The FVIID mutation originally reported in Beagles [17] has now been reported in over a dozen breeds. It was, therefore, unsurprising to find genetically-susceptible and carrier dogs in our mixed-breed population. Although there were slightly more dogs that were homozygous for the alternate/mutant FVIID allele (n = 65) compared to those homozygous for the alternate/mutant HUU allele (n = 57), the carrier rate (0.846% vs 4.45%) and allele frequency (0.614% vs 2.39%) were much lower in FVIID compared to HUU, respectively. Amongst the dogs shown to be homozygous for the alternate/mutant FVIID allele, we were able to identify recent Beagle ancestry in only 13 of the dogs. A very low number of the other breeds previously-reported to carry the alternate/mutated FVIID allele were observed, such as American Foxhounds and Miniature Schnauzers. But for 50 of the 65 homozygous FVIID dogs, no breed was identified in their recent ancestry that explained the presence of the alternate/mutated allele; in a further three dogs, only one great-grandparent breed was identified that could have contributed the alternate/mutant allele. Again, this could be due to the algorithm failing to detect very small contributions of Beagle or other known FVIID-affected breeds from further back than the great-grandparent level, but it also implies that more breeds will eventually be shown to carry the alternate/mutant FVIID allele. An extremely diverse background of breeds with no discernable trends were observed in this group of FVIID mutant homozygous dogs, making it difficult to predict which breeds might be more likely to actually be carrying the alternate/mutant FVIID allele, but also hinting that many as-yet-unidentified breeds may carry this allele. Given the extremely broad genetic background of breeds carrying the alternate/mutant FVIID allele in their populations, it is judicious to consider FVIID for any mixed-breed dog. Of particular concern with this disease is its widely variable presentation: bleeding is reported as being mild to moderate in Beagles [17] but severe in the Alaskan Klee Kai [18]. Clinical signs are typically noted after surgical procedures, so FVIID should be considered in all mixed-breed dogs when clotting issues are relevant, even though Beagle ancestry may not be discernable. Proactive genetic testing of all mixed-breed dogs would certainly be advantageous prior to surgical interventions, but, at a minimum, practitioners must keep inherited FVIID on their list of rule-outs when they encounter excessive bleeding in mixed-breed dogs. Again, this mutation, along with many others, is increasingly available as an option in genetic screening panel testing, making it cost effective to suggest to owners of mixed-breed dogs.

Conversely, the other three disorders were statistically significantly less likely to occur across all measures (allele frequency, carrier frequency, and genetically-susceptible frequency). While a small number of PFKD carriers (n = 19) were observed, CYST and MYC were vanishingly rare in this cohort. The low number of PFKD carriers, and absence of genetically-susceptible dogs, was surprising given the diverse breeds reported to carry this mutation (American Cocker Spaniels, English Springer Spaniels, and Whippets); in addition to simply having a lower frequency, this could also be due to the fact that these breeds do not contribute as much to the North American mixed-breed population. While these diseases should obviously still be considered on a diagnostic rule-out list when clinically relevant, the present study indicates that mixed-breed dogs are statistically protected from PKFD, CYST, and MYC.

This data set also shows that mixed-breed dogs can even have more than one genetic mutation, although this is quite rare. This indicates that it is still possible for a mixed-breed patient to suffer from more than one Mendelian genetic disorder, a fact the practitioner should keep in mind.

In general, genetic disease occurs more frequently among purebred dog populations than mixed-breed dog populations, which typically experience less purposeful selection and inbreeding. However, given that some genetic disorders, such as HUU, stem from mutations that likely developed long before the breeds were distinguished, it is imperative that genetic disorders still be considered in the evaluation and diagnosis of disease in mixed-breed dogs. Genetic disorders should not be ruled out by a practicing veterinarian simply because the dog is not a purebred individual. Hundreds of Mendelian mutations are now published in dogs, and companies are beginning to provide testing platforms that examine dozens of these. As data become available for these tests, they will allow further refinement of the list of genetic diseases that are most common in mixed-breed dogs. The present data set will also provide a benchmark in time against which future studies can compare allele frequencies in similar populations to see if these are changing.

## Conclusions

By using a large, continent-spanning population, this study has shown that dogs of mixed-breed origins, including dogs with very complex ancestral backgrounds, can be affected by genetic diseases. Of the five diseases examined in the present study, HUU and FVIID should be readily considered by the practitioner, when clinically appropriate, even for mixed-breed dogs.

## Supporting information

S1 Table Result strings for each dog. Eight called great-grandparent breeds are shown, along with each dog’s genotypes/results for the five disease genetic tests. When any individual, or multiple, great-grandparent breed(s) do not reach an appropriate confidence threshold, they are not reported on pedigree trees. Such lower-confidence breeds are designated as “Mixed”, rather than reporting an uncertain breed.
(XLSX)

S2 Table P-Values for pairwise Fisher tests. P-values are reported for all pairwise Fisher tests, with Bonferroni-Holm correction for multiple comparisons; p ≤ 0.05 was considered significant. Within each column, the letter indicates this value is statistically significantly different from all other different letters in that column.
(XLSX)
